# Identification of a missense *ARSA* mutation in metachromatic leukodystrophy and its potential pathogenic mechanism

**DOI:** 10.1002/mgg3.1478

**Published:** 2020-09-01

**Authors:** Liyuan Guo, Bo Jin, Yidan Zhang, Jing Wang

**Affiliations:** ^1^ CAS Key Laboratory of Mental Health Institute of Psychology Chinese Academy of Sciences Beijing China; ^2^ Department of Psychology University of Chinese Academy of Sciences Beijing China; ^3^ Department of Neurology Children's Hospital of Nanjing Medical University Nanjing Jiangsu China

## Abstract

**Background:**

Metachromatic leukodystrophy (MLD) is a rare inherited lysosomal disorder caused by mutations in *ARSA*. The biological processes of MLD disease caused by candidate pathogenic mutations in the *ARSA* gene remain unclear.

**Methods:**

We used whole‐exome sequencing (WES) and Sanger sequencing to identify the pathogenic mutation in a Chinese family. Literature review and protein three‐dimensional structure prediction were performed to analyze the potential pathogenesis of the identified mutations. Overexpression cell models of wild‐type and mutated *ARSA* genes were constructed. The accumulated sulfatides and expression profiles in the cell models were detected, and a series of bioinformatics analyses were carried out to compare the biological changes caused by the candidate pathogenic mutations.

**Results:**

We identified an *ARSA* c.925G>A homozygous mutation from a Chinese late‐infantile MLD patient, the first report of this mutation in East Asia. The literature and protein structure analysis indicated that three types of mutations at c.925G (c.925G>A, c.925G>T, c.925G>C) were pathogenic. The overexpression of wild‐type or mutated *ARSA* genes influenced the accumulation of sulfatides. The co‐expression modules in the mutated cell models were constructed by genes related to calcium signaling and vesicle transport.

**Conclusion:**

Our results identified a pathogenic mutation, *ARSA* homozygosity c.925G>A, from a Chinese MLD family. The pathogenic mechanism of the *ARSA* mutation in MLD was identified, which may suggest new approaches to diagnosis and treatment.

## Graphical abstract

We identified an ARSA c.925G>A homozygous mutation from a Chinese late‐infantile MLD patient, the first report of this mutation in east Asia. Overexpression cell models of wild‐type and mutated ARSA genes were constructed to compare the biological changes caused by candidate pathogenic mutations. The results indicate that the mutations at c.925G cause comprehensive molecular changes related to energy metabolism, ion binding, vesicle transport, and transport.

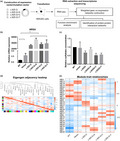

## 1 INTRODUCTION

Metachromatic leukodystrophy (MLD) is a rare inherited lysosomal disorder caused by recessive gene mutations in *ARSA* (OMIM: 607574) and *PSAP* (Kihara, 1982; Kihara, Fluharty, O'Brien, & Fish, 1982; van Rappard, Boelens, & Wolf, 2015; Schielen, Kemper, & Gelb, 2017). There are five allelic forms of MLD (MIM ID: 250100), including late‐infantile, juvenile, adult, partial cerebroside sulfate sulfatase deficiency, and pseudoarylsulfatase A deficiency (Kihara, 1982; Kihara et al., 1982); and two nonallelic forms, including metachromatic leukodystrophy due to saposin B deficiency (MIM ID: #249900) and multiple sulfatase deficiency or juvenile sulfatidosis (MIM ID: #272200), a disorder that combines the features of mucopolysaccharidosis with those of metachromatic leukodystrophy. Although a series of potential therapies, such as hematopoietic stem cell transplantation (Sessa et al., 2016), enzyme replacement therapy (Chen, Gill, Shaw, Ouvrier, & Troedson, 2016), and gene therapy (Meneghini et al., 2016) have been explored, currently there is no curative treatment for this disease.

The most common type of MLD disease is an autosomal recessive inherited lysosomal disorder caused by mutations in the *ARSA* gene, located on the chromosome 22q13.33, resulting in a deficiency of the enzyme arylsulfatase A. The low activity of arylsulfatase A results in the accumulation of sulfatides in the central and peripheral nervous systems, leading to demyelination (van Rappard et al., 2015). To date, more than 200 *ARSA* allele types have been reported as *ARSA*‐causative variants (Cesani et al., 2016).

In the current study, we report a Chinese family with a 2‐year‐old boy who could not sit or walk independently. By whole‐exome sequencing (WES) technology we identified a missense mutation in exon 5 of the *ARSA* gene (c.925G>A homozygous mutation) in the proband that supported the clinical diagnosis of MLD. This is the first report of this mutation in East Asia. A systematic literature review and bioinformatics analyses were carried out to predict its pathogenicity. Furthermore, transcriptome profiling of overexpression cell models of the wild‐type and mutated *ARSA* gene was analyzed to explore the potential molecular pathogenesis of the identified mutation.

## 2 METHODS

### 2.1 Ethical compliance

The current study was approved by the Ethics Committee (No. 201801001‐1) of the Children's Hospital Affiliated with Nanjing Medical University (Nanjing, China). Prior to the study, written informed consent for genetic tests and publication of the case details were obtained from all adult participants and the parents or the legal guardians of the children involved in the study.

### 2.2 Mutation analysis

Peripheral blood samples were obtained from the patient and members of his family. WES was performed on all family members and then variant calling was performed with reference to the UCSC hg19 reference genome (http://genome.ucsc.edu/) (Rhead et al., 2010). Single‐nucleotide polymorphisms (SNPs) and small insertions or deletions (indels) were annotated by ANNOVAR (2015Dec14) (Wang, Li, & Hakonarson, 2010). Short insertions or deletions (indels) altering coding sequences or splicing sites were also identified by GATK (3.3.0). Online tools including Polymorphism Phenotyping version 2 (PolyPhen‐2, http://genet​ics.bwh.harva​rd.edu/pph2/) (Adzhubei, Jordan, & Sunyaev, 2013), Sorting Intolerant from Tolerant (SIFT, http://sift.jcvi.org/; a score less than 0.05 indicates it is deleterious) (Ng & Henikoff, 2003), and MutationTaster (http://www.mutat​ionta​ster.org/) (Schwarz, Rodelsperger, Schuelke, & Seelow, 2010) were used to evaluate whether the amino acid substitutions affected protein function. Variants were identified as nonpathogenic and removed if they fit any of the following criteria: (1) variants with an allele frequency greater than 5%; (2) variants located in introns and not affecting splicing; (3) synonymous variants not affecting splicing; or (4) variants located in the 5’ or 3’ untranslated regions. Then we reviewed the functions and associated OMIM phenotypes of these genes (https://omim.org/). Locus‐specific primers used for PCR amplification and direct sequencing were designed (GenBank: NM_000487.5 and NP_000478.3) and then Sanger sequencing of the PCR products was performed to validate the potentially disease‐causing variants (completed in Oumeng V Medical Laboratory Co., Ltd.) (Yuen et al., 2015). A systematic literature review was performed to obtain published *ARSA* c.925 mutations in MLD (Figure S1). Amino acid conservation of the ARSA protein in different species was performed by MEGA5 and DNAMAN software. The effect of the amino acid changes in *ARSA* at p.E309 was predicted by the web server SWISS‐MODEL (https://www.swiss​model.expasy.org).

### 2.3 Overexpression cell models of the mutated *ARSA* gene

The human wild‐type *ARSA* cDNA (cloned in the pCMV6 plasmid) was purchased from OriGene (Cat. No. RC204319, OriGene, USA). The c.925G mutations (c.925G>A, c.925G>T, and c.925G>C, as shown in Figure S2) were introduced into the *ARSA* plasmid by Site‐Directed Mutagenesis Kit (Cat. No. E0552 s, NEB, USA). The sequences of the mutated cDNA vectors were confirmed using an ABI3500 sequencer (Applied Biosystems Inc.). The *ARSA* gene sequence was obtained from the NCBI database (GenBank: NM_000487.5 and NP_000478.3). HEK293 cells (CRL‐1573, ATCC) were cultured in high‐glucose DMEM (Cat. No. 11965092, Life Technologies) supplemented with 10% FBS at 37°C and 5% CO_2_. Cells were seeded 24 hours before transfection and were collected 24 hours after the transfection for qPCR and RNA‐seq. The transient transfection was performed by FuGENE HD transfection reagent (Cat. No. E2311; Promega) and the ratio of the efficiency of transfection was approximately 70%. The ratio of the efficiency of transfection was detected by the fluorescence of HEK293 cells transfected with pCMV6‐GFP fusion plasmid (Figure S3).

### 2.4 Sulfatides concentration determination

HEK293 cells were seeded 24 hours before transfection and were collected 48 hours after the transfection. Then, the cells were lysed in RIPA lysis buffer and the lysates were centrifuged at 13000 g for 5 min. The supernatant was used for protein concentration measurements and sulfatides concentration determination. The sulfatides concentration of the HEK293 cells was detected by a double antibody sandwich ELISA kit (Cat. No. A098069, Fusheng). Briefly, the cell supernatant was diluted five times and added to ELISA coated wells, and after incubation at 37°C for 30 min, the conjugate reagent was added after washing and then the plate was incubated at 37°C for another 30 min. The TMB substrate and stop buffer were added in sequence. Finally, the absorbance was measured at 450 nm by a microplate reader (Infinite F50, TECAN). The sulfatides concentration of the sample was calculated by a standard curve (set up by standard samples at concentrations of 12.5, 25, 50, 100, and 200 pg/ml), and standardization of the sulfatides amount was performed by dividing it by the amount of total protein in the lysates.

### 2.5 Transcriptome profiling analysis

RNA sequencing of overexpression cell models and subsequent bioinformatics analysis were completed by the Oumeng V Medical Laboratory. RNA sequencing was performed using the same protocol with poly‐A selection of mRNA following the manufacturer's instructions (Illumina TruSeq). Paired‐end 150 bp sequencing, which was performed on Illumina HiSeq 2000 instruments, generated more than 6G clean bases data for each sample. The raw reads were quality checked using FastQC. Low‐quality bases were trimmed with Trimmomatic (parameters TRAILING: 3 and SLIDINGWINDOW: 4:15). HISAT2 was used to compare clean reads to the reference genome (GRCh37). Then, sequence mapping data (SAM) were converted into bam files and sorted according to the mapped chromosomal location using SAMtools.

The software StringTie was used to calculate the gene expression and outputted the standardized TPM format expression data of all genes in each sample. Expression profiling of all samples was utilized as input data to construct co‐expression networks and obtain a co‐expression gene module using the R package of WGCNA (Langfelder & Horvath, 2008). We calculated the Pearson's correlation between modules and cell treatments to identify the relevant module. In each co‐expression module, the genes were ranked according to the four ranking methods in Centiscape2.2 (Degree, Stress, Closeness, and Radiality), and the overlaps of the first 30 genes of each ranking method were defined as the hub genes. The modules were defined as key modules when they had a correlation >0 and *p* value<0.05 with at least one of the four types of cells (c.925G, c.925G>A, c.925G>T, c.925G>C).

The significantly enriched biological processes and pathways of the genes in the key modules were analyzed using the Gene Functional Classification Tool (DAVID) v6.8, (Huang et al., 2007). The online tool Metascape was used to perform annotations of the hub genes from the Gene Ontology (GO) database and Kyoto Encyclopedia of Genes and Genomes (KEGG) database. The gene lists from the selected modules were used to build a protein–protein interaction (PPI) network based on BioGrid6, InWeb_IM7, and OmniPath database in the online tool Metascape. We input the correct species (*H. sapiens*) and chose “Express Analysis” for analysis. Moreover, if the network contains between 3 and 500 proteins, the “Molecular Complex Detection” (MCODE) algorithm was applied to identify densely connected network components.

## 3 RESULTS

### 3.1 Clinical and genetic findings

**FIGURE 1:**
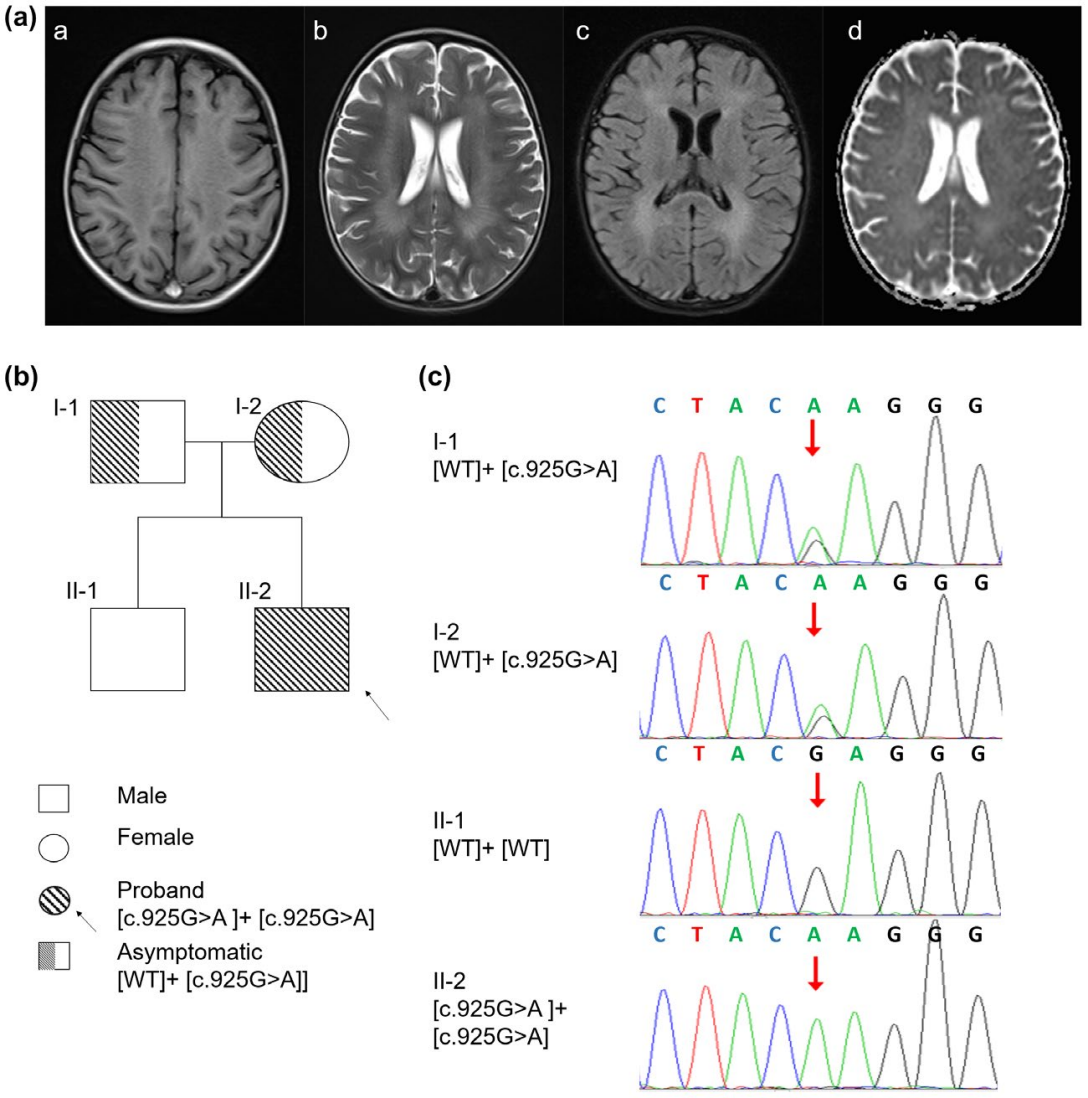
Proband, family, and mutation. (A) MRI from the proband (II‐2). Magnetic resonance imaging (MRI) shows symmetrical deep lesions located in periventricular white matter, which was low signal in T1WI (a), high signal in T2WI (b), low signal in in T2WI (c) and ep2d (d) from the proband (II‐2). (B) Pedigree of the family with MLD patients. The proband was shown in the second generation with the numbers II‐2. The parents of proband are first generation with the number I‐1 and I‐2. The healthy older brother of proband is in the second generation with the number II‐1. (C) Mutational analysis of the arylsulfatase A (*ARSA*) gene. Genotypes of the proband showed a homozygous c.925G>A mutation, and those of the parents showed a heterozygous c.925G>A mutation. His healthy brother did not inherit this mutation. Nucleotide numbers are derived from cDNA *ARSA* sequences, GenBank accession numbers: NM_000487.5 and NP_000478.3.

The proband, a 27‐month‐old boy, was unable to stand or walk independently. He showed decreased lower limb muscle strength, decreased muscle tension, and electrophysiological changes in multiple peripheral neurogenic lesions on electromyography. Abnormal white matter symmetry signals in bilateral cerebral hemispheres were observed on MRI images of the proband (Figure 1A). He was the second boy of a healthy, nonconsanguineous couple (Figure 1B). The results of the WES tests and quality controls for all family members are shown in Tables S1–S3. According to the American College of Medical Genetics and Genomics (ACMG) recommendations, which provides interpretative categories of sequence variants and an algorithm for interpretation (Richards et al., 2015), a missense mutation, c.925G>A in exon 5 of the *ARSA* gene (Figure 1C, Table S4) was identified as a candidate pathogenic locus. Genotypes of the proband showed a homozygous c.925G>A mutation, and those of the parents each showed a heterozygous c.925G>A mutation (I‐1, I‐2, Figure 1C). His healthy brother did not inherit this mutation (II‐1, Figure 1C).

### 3.2 *ARSA* mutation (c.925G>A, c.925G>T, c.925G>C) function

**FIGURE 2:**
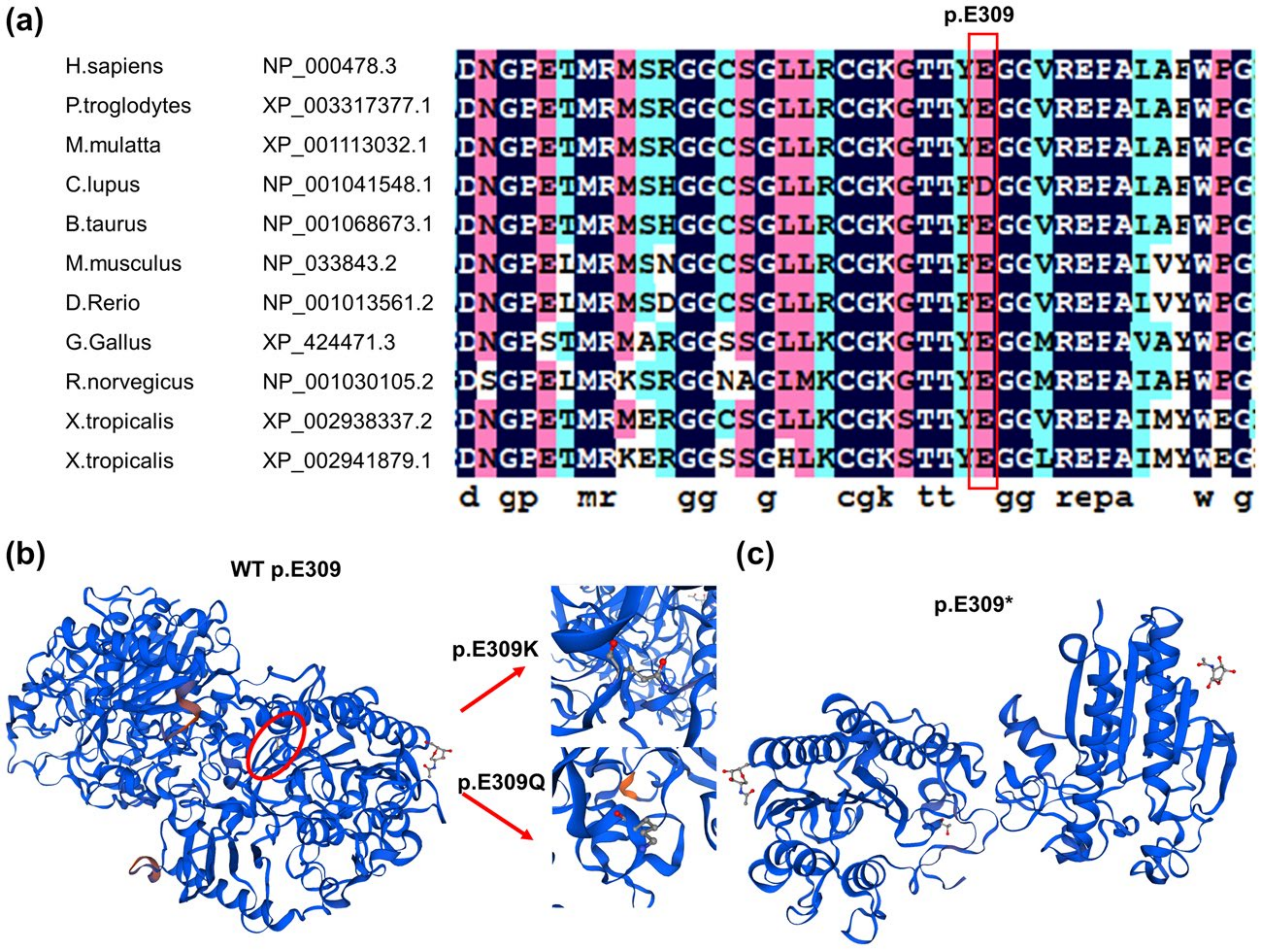
Multiple sequence alignment (MSA) and 3D structure of ARSA. (a) Multiple sequence alignment showing the sequence alignment of a specific amino acid, and its conservation in other *ARSA* orthologs (across different species). Nucleotide numbers are derived from cDNA *ARSA* sequences, GenBank accession numbers: NM_000487.5 and NP_000478.3. (b) Conformational changes induced by the p.E309K and p.E309Q missense mutation in the ARSA protein. (c) Conformational changes induced by the p.E309*, this mutation results in the early termination of codons and truncated proteins.

c.925G>A (p.E309 K) is highly conserved in multiple species sequences, suggesting its structural and functional importance (Figure 2a). This mutation was predicted to affect the protein features and be disease‐causing by SIFT, PolyPhen2, and MutationTaster (Table S4). The 3D structural model analysis showed that the glutamate at position 309 took part in the formation of a disulfide bond. Hence, three missense mutations (c.925G>A, c.925G>T, c.925G>C) caused amino acid changes (p.E309 K, p.E309* and p.E309Q) and truncated the protein, altering the local charge to prevent the correct positioning of the sulfate group of the substrate (Figure 2). In previous studies, mutations in this site (c.925G>A, c.925G>T, c.925G>C) have been reported to cause late‐infancy MLD. As shown in Table 1, one of the 10 reported patients with MLD disease had adult MLD, and one was diagnosed with juvenile MLD; four patients had c.925G>A homozygous mutations (including the proband in this study) (Cesani et al., 2016; Onder, Sinici, Mujgan Sonmez, Topcu, & Ozkara, 2009), and other patients had heterozygous mutations at this site (Biffi et al., 2008; Cesani et al., 2009; Chen et al., 2018; Grossi et al., 2008; Kreysing et al., 1993).

**TABLE 1:** Reported summary of characteristics about the *ARSA* c.925G mutation Patients.

Pt No.	Fam	MLD variant	Nat/ Ethnicity	Gender	Age		Symptoms at onset	Genotype	Enzymatic activity	References
					Onset	Death				
1	—	LI	Caucasian	F	1y06m	—	Walking difficulties, dysarthria, and spasticity	[c.925 G>A] + [c.925 G>A]	6%	Cesani et al. (2016)
2	—	A	Caucasian	M	40y	—	Peripheral neuropathy	[c.869 G>A] + [c.925 G>C]	—	Cesani et al. (2016)
3	—	LI	Chinese	F	4y	—	Psychomotor deterioration, motor regression, Dysarthria, Symmetrical deep WM abnormalities	[c.925 G>A] + [c.427 T>C]	—	Chen et al. (2018)
4	—	LI	Chinese	F	0y07m	—	Motor retardation, Peripheral neuropathy, Symmetrical deep WM abnormalities	[c.925 G>A] + [c.302 G>T]	—	Chen et al. (2018)
5^a^	Sibling	LI	European	M	01y06m	—	Walking difficulties, Pain attacks, Peripheral neuropathy	[c.304delC] + [c.925 G>A]	12%	Kreysing et al. (1993)
6^a^	—	normal	European	F	—	—	Healthy	[WT] + [c.925 G>A]	—	Kreysing et al. (1993)
7^a^	Sibling	normal	European	M	—	—	Healthy	[WT] + [c.925 G>A]	—	Kreysing et al. (1993)
8	—	J	European	—	—	—	Progressive and profound motor deficit	[c.418 C>G] + [c.925 G>A]	0%	Biffi et al. (2008)
9^b^	Sibling	LI	Chinese	M	01y05m	—	Walking difficulties, Peripheral neuropathy	[c.925 G>A] + [c.925 G>A]	—	This study
10^b^	—	normal	Chinese	M	—	—	Healthy	[WT] + [c.925 G>A]	—	This study
11^b^	—	normal	Chinese	F	—	—	Healthy	[WT] + [c.925 G>A]	—	This study
12	—	LI	Italian	—	02y00m	—	Spastic paraparesis Ataxia, Mental deterioration, Symmetrical deep WM abnormalities	[c.925 G>T] + [c.59 C>A]	—	Grossi et al. (2008)
13	—	LI	Turkish	M	01y06m	03y06m	Difficulty in walking, intentional tremor, nystagmus, spontaneous contraction at extremities and wheezing.	[c.925 G>A] + [c.925 G>A] [c.1178 C>G] + [c.1178 C>G]	—	Onder et al. (2009)
14	—	LI	Turkish	F	02 y06m	04y00m	Difficulty in walking, positive bilateral Babinski, tremor in hands and mild spasticity	[c.925 G>A] + [c.925 G>A] [c.1178 C>G] + [c.1178 C>G]	—	Onder et al. (2009)

Note

Nucleotide numbers are derived from cDNA ARSA sequences, GenBank accession numbers: NM_000487.5 and NP_000478.3. Mutations are described according to current mutation nomenclature guidelines (http://www.hgvs.org/mutnomen; den Dunnen and Antonarakis, 2000), ascribing the A of the first ATG translational initiation codon as nucleotide +1.

Abbreviations: A, adult; F, Female; Fam, familiarity; J, juvenile; LI, late infantile; M, Male; m, month; Presymp, presymptomatic; Pt, patient; Sibs, sibling; y, year. The symbol “–” indicates data not available.

^a^ DNA was isolated from fibroblasts of the patient No. 5, his mother No.6, and his brother No.7.

^b^ DNA was isolated from peripheral blood of the patient No. 9, his father No.10 and his mother No.11.

### 3.3 Amount of accumulated sulfatides in overexpression *ARSA* cell models

**FIGURE 3:**
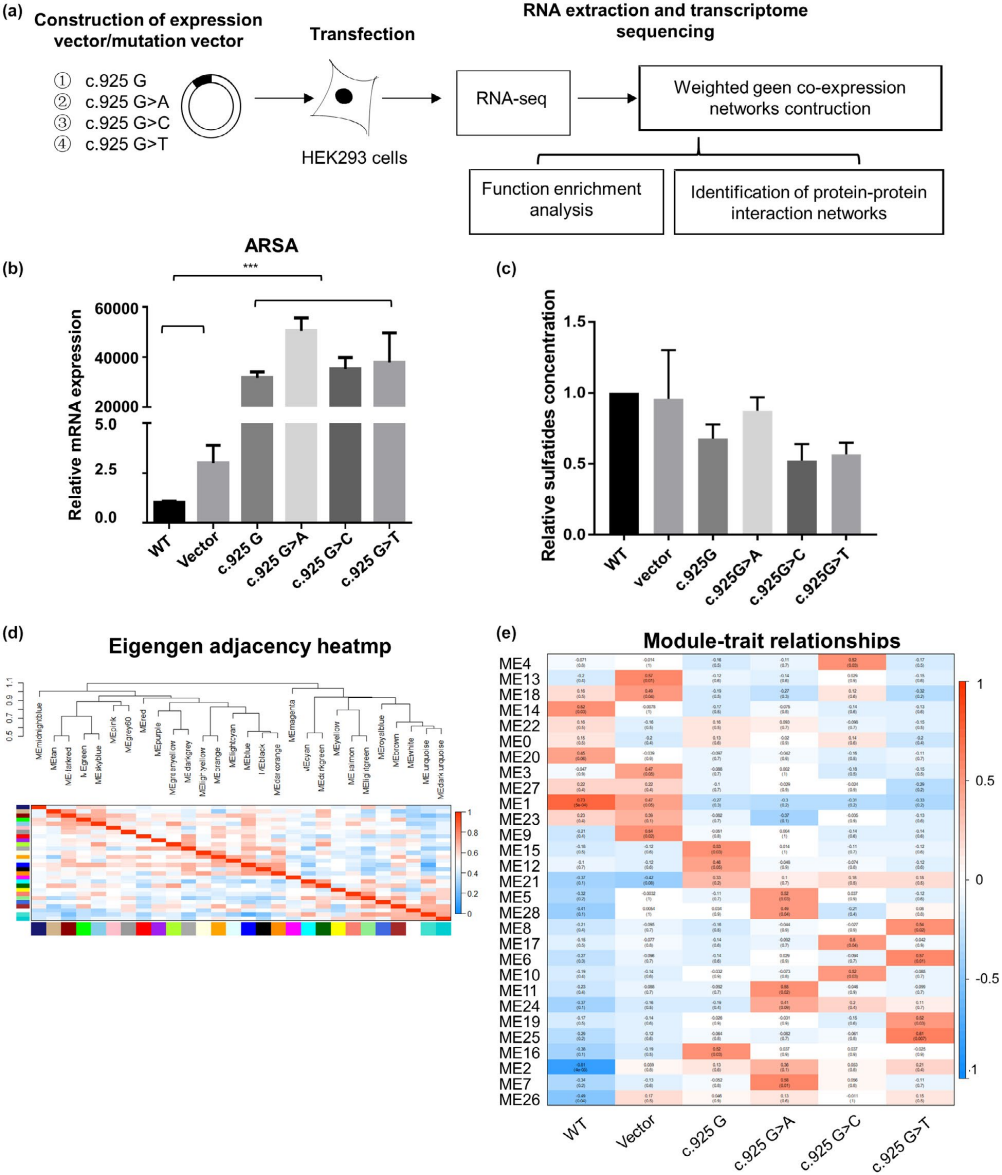
Transcriptomic analysis in overexpression cell models of wild‐type and mutated *ARSA* gene. (a) Construction of overexpression cell models of wild‐type and mutated *ARSA* gene. (b) Relative mRNA expression of *ARSA* gene, GAPDH was used as a loading control. *p < 0.05 in independent Student's *t*‐test, ***p < 0.001 in independent Student's *t*‐test (n = 3). (c) Sulfatides concentration in wild‐type cells and *ARSA* gene overexpression cell models. The standardization of sulfatides amount was performed by dividing it into the amount of total protein of the lysates. No significant difference was found in independent Student's *t*‐test (n = 3). (d) Hot Map of Characteristic Gene Adjacency in Characteristic Gene Network. Each row and column corresponds to a characteristic gene (marked with the same color). In heatmap, red denotes high adjacency (positive correlation) and blue denotes low adjacency (negative correlation), as shown in the color legend. (e) Characteristic gene module‐trait association of each module. Each row corresponds to a module characteristic gene, and each column corresponds to a cell type (trait). Each cell contains Pearson correlation coefficients (numbers outside parentheses) and associated *p* values (numbers inside parentheses). According to the color legend, color coding is carried out by correlation. Red indicates positive correlation and blue indicates a negative correlation.

To further elucidate the pathogenesis of the mutations of p.E309, we built overexpression cell models of wild‐type and mutated *ARSA* gene. Such a cell mutation model approach has been successfully applied to several rare disease studies (Galla et al., 2013). Mock vector (Vector), wild‐type *ARSA* cDNA plasmid (c.925G), and missense mutated *ARSA* cDNA plasmid (c.925G>A, c.925G>T, c.925G>C) were transfected into HEK293 cells. RNA‐seq and consequent bioinformatics analyses were performed on the untreated and transfected cells (Figure 3a). As validated by RT‐qPCR, the expression level of *ARSA* exceeded 20,000‐fold of the wild‐type and the mutated *ARSA* overexpression cell models compared with the vector (Figure 3b). Compared with the nontransfected HEK293 cells and the cells transfected with mock vectors, the concentration of sulfatides (the substrate of arylsulfatase A) in wild‐type *ARSA* overexpression cells was decreased and the decreasing tendency was weakened in the c.925G>A mutated *ARSA* overexpression cells. The sulfatides concentration in the c.925G>C and c.925G>T mutated *ARSA* overexpression cells was even lower than in the wild‐type *ARSA* overexpression cells (Figure 3c). The differences among the groups were not statistically significant.

### 3.4 Co‐expression networks in the overexpression *ARSA* cell models

A description of the expression profiling in each cell model is shown in Table S5. After the WGCNA analysis, 28 co‐expression modules were identified. The interactions among these 28 co‐expressed modules (reflected by the connectivity of the eigengenes) are shown as a heatmap of the module eigengene adjacency (Figure 3d). Then, the module‐trait relationships with all traits were calculated by correlating the module's eigengenes to the traits of interest (Figure 3e) and were used for the selection of the modules for downstream analysis. Fourteen modules were significantly positively correlated with four *ARSA* overexpression models (c.925G, c.925G>A, c.925G>T, c.925G>C).

**FIGURE 4:**
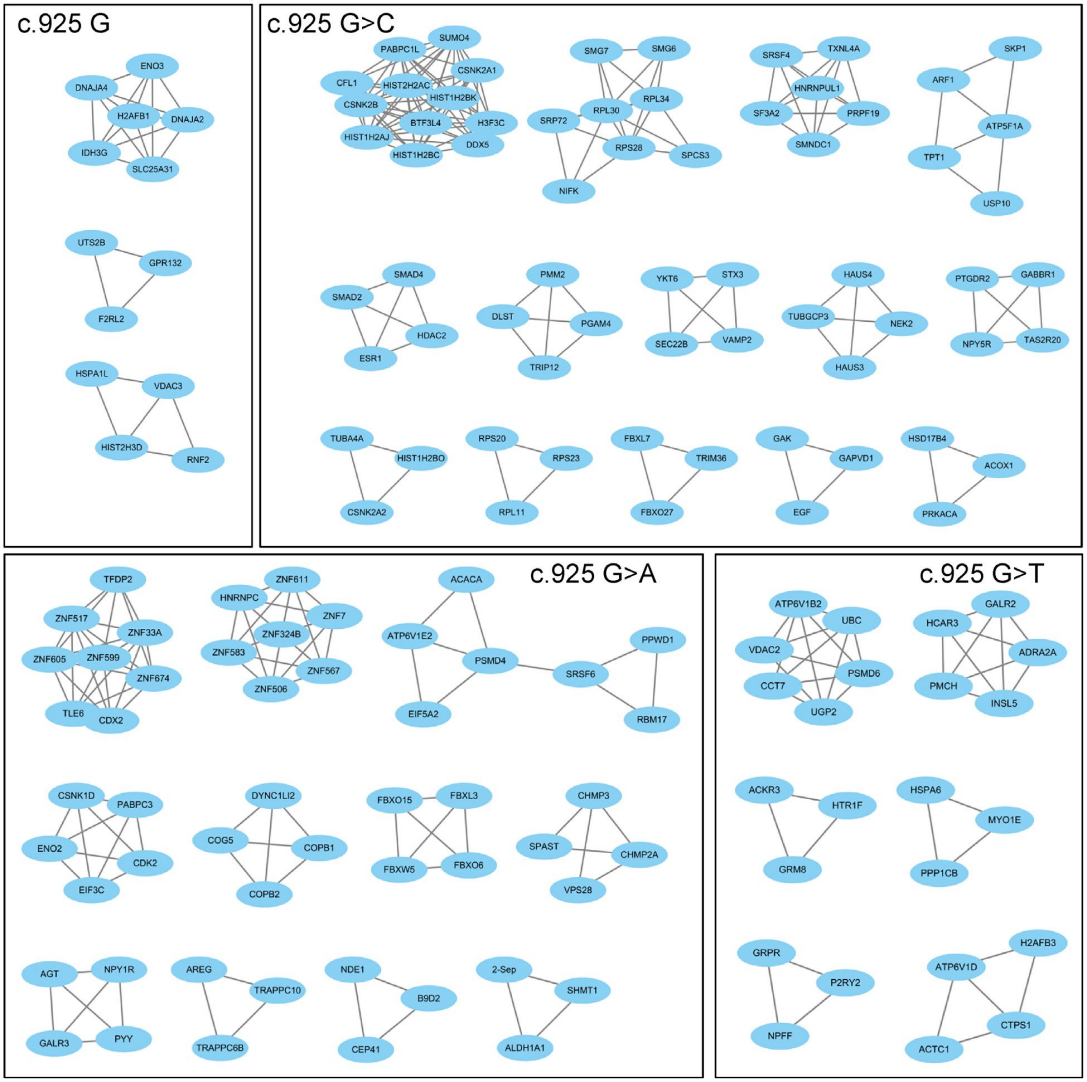
Protein–protein interaction networks in the overexpression cell models of wild‐type and mutated *ARSA* gene.

Genes in the modules related to the c.925G>A mutated cell model showed enrichment for functions related to calcium ion‐dependent vesicle fusion, intra‐Golgi vesicle‐mediated transport, ion‐binding, and ion transport. In the c.925G>C mutated cell model, the enriched pathways were associated with the mitochondrial inner membrane, vesicle‐mediated transport, and calcium binding. In the c.925G>T mutated cell model, enrichment in the carbohydrate metabolism process pathway was found (Table 2). The functions of the hub genes in the mutation related modules further supported the above results. The hub genes identified in the c.925G>A module were related to Golgi vesicle‐mediated transport (such as *TRAPPC6B*, *COG5*), cell junctions (*SUN1*), and ion‐binding related genes (such as *ZNF583* and *TRAPPC6B*). The hub genes identified in the c.925G>C module were related to the mitochondrial inner membrane (*LRMP* and *SMIM24*), vesicle‐mediated transport (*F5*, *LRMP*, *CALCR*, *AAK1*, etc.), and calcium binding (*F5* and *CALCR*). The hub genes identified in the c.925G>T module were enriched in the carbohydrate metabolism process pathway (*BCL2L1* and *PSMC5*) (Table S6). In addition to the hub genes, the PPI analysis also showed similar results (Figure 4), which suggested more interacting protein complexes in the mutated cell models than in the wild‐type cell model.

**TABLE 2:** Functional enrichment analysis.

Sample	Enrichment cluster	Enrichment score
c.925 G	Protease inhibitor	2.54
	Intracellular signal transduction	1.33
c.925 G>A	Calcium ion‐dependent vesicle fusion	1.86
	intra‐Golgi vesicle‐mediated transport	1.79
	Hormone	1.55
	Cell junction	1.48
	meta ion‐binding	1.47
	Ion transport	1.31
c.925 G>C	Ankyrin repeat‐containing domain	1.69
	Mitochondrion inner membrane	1.53
	vesicle‐mediated transport	1.45
	integral component of membrane	1.39
	RNA polymerase II transcription factor activity, ligand‐activated sequence‐specific DNA binding	1.38
	calcium‐binding	1.33
	phosphatidylinositol phosphorylation	1.32
c.925 G>T	carbohydrate metabolic process	1.51

Significantly enriched functional clusters (defined as Enrichment Score of >1.3) as determined by DAVID functional annotation clustering analysis.

## 4 DISCUSSION

We reported a boy with MLD‐like traits. The genetic detection showed that the child carried an *ARSA* c.925G>A homozygous mutation. This mutation was first reported by the Kreysing research team, and they found that the c.304del C and c.925G>A mutation sites were pathogenic, while a heterozygous mutation of c.925G>A had no typical clinical manifestation in the parents (Kreysing et al., 1993). It has been found that c.925G>A is a complex heterozygous variant with a variety of mutation sites, leading to the occurrence of MLD disease (L. Chen et al., 2018). The homozygous mutation site c.925G>A was first found by Onder in 2 Turkish patients from different families (Onder et al., 2009). In the current study, we report the first homozygous mutation of c.925G>A in a child from East Asia.

The c.925G encodes glutamate at position 309 of the ARSA protein (p.E309), which is highly conserved among different species (Figure 2a). This indicates that amino acid changes at this position would significantly affect the structure and function of the ARSA protein. The crystal structure of human arylsulfatase A shows that the core of the enzyme is composed of two β‐pleated sheets. The major β‐pleated sheet is formed by 10 β‐strands and it is sandwiched between three α‐helices on one side and four on the other (Lukatela et al., 1998). In our study, the 3D structural model analysis showed that p.E309 K was involved in the formation of a disulfide bond.

A previous study reported the biochemical characterization of the *ARSA* mutation p.E309 K and confirmed the pathogenic nature of the mutation by in vitro mutagenesis of the *ARSA* gene and transient transfection to Chinese Hamster ovary cells (Ozkan & Ozkara, 2016). Previous studies have described other pathogenic mutations (p.K304R, p.K304 N, p.T306 M, p.Y308H, p.E309Q, p.E309*, p.G310D, p.G311S, and p.R313Q) in the disulfide bond region of *ARSA* (Cesani et al., 2016), which may cause MLD. The sulfatides concentration in the mutated *ARSA* overexpression cells also indicated that the mutated *ARSA* gene (c.925G>A, c.925G>T, c.925G>C) may impact sulfatide metabolism. To sum up, our results suggested that the p.E309 (p.E309 K, p.E309Q, p.E309*) mutation may lead to a change in the spatial steric hindrance and conformation of the *ARSA* structure, which may change the morphology of the arylsulfatase A protein and impair sulfatide metabolism.

Systematic bioinformatics analyses of expression profiling in *ARSA* overexpressing cells provided more clues about the complex biological processes that were impacted by the mutations. By co‐expression network analysis, we obtain gene modules influenced by mutations at c.925 of *ARSA*, and the subsequent hub genes analysis, PPI analysis and functional enrichment analysis indicated that these modules may mostly impact the biological function of calcium signaling and vesicle transport (Figure S4). Perturbations of calcium signaling have been reported to contribute to demyelination and lysosomal storage (Mu, Fowler, & Kelly, 2008; Patil & Maegawa, 2013), and in our study, more than one of the mutation signature modules were enriched in calcium‐related functions. This suggests potential roles of calcium signaling in MLD pathogenic cascades. Vesicle transport‐related genes were also dysregulated in the mutated cell models, showing the importance of vesicle transport in cell catabolism (Platt, Boland, & van der Spoel, 2012), and these *ARSA* mutations may induce lysosomal dysfunction by impacting vesicle transport.

In conclusion, we identified a late infantile metachromatic leukodystrophy patient carrying a c.925G>A homozygous mutation in the *ARSA* gene. Functional analysis of the mutation in the current study provides a potential pathogenic mechanism of the *ARSA* mutation in metachromatic leukodystrophy and suggests possible new strategies for diagnosis and treatment.

## CONFLICT OF INTEREST

The authors declare that there is no conflict of interest.

## AUTHOR CONTRIBUTION

LG designed and performed the analysis. BJ contributed to patient care and diagnosis. YZ performed experiments. JW designed the analysis drafted the manuscript.

## Supporting information

Supplementary Material
