# Supplementary material for: Identification of a missense ARSA mutation in metachromatic leukodystrophy and its potential pathogenic mechanism
Source: Mol Genet Genomic Med. 2020 Sep 1;8(11):e1478. doi: 10.1002/mgg3.1478 (PMC7667344; doi:10.1002/mgg3.1478)
Supplement: Supplementary file 1 — Supplementary Material [file MGG3-8-e1478-s001.pdf]

Supplementary Table 1. Quality of the Whole exome sequencing in this study.

Statistic	Sample			
	I-1	I-2	II-1	II-2
Raw Bases (bp)	17642855700	19467058800	21535325400	24746674200
Clean Bases (bp)	16399403923	18545867724	20188868376	23250333561
Clean Q30 (%)	92.48	94.9	94.93	94.64
Clean Max GC/AT Bias Rate (%)	7.69/6.88	7.30/7.44	7.71/6.60	7.60/6.91
Clean GC Content (%)	51	50	51	51
Clean Reads Rate (%)	92.95	95.27	93.75	93.95
Capture Efficiency (%)	62.25	57.18	63.78	63.1
Clean Reads Mapped Rate (%)	99.95	99.93	99.97	99.97
Insert Size of Clean Reads (bp)	256	267	242	244
Insert Size Std Dev	59.92	68.05	52.55	55.62
Duplication Clean Reads Rate	2.33	2.62	2.4	2.66
Uniq Reads Rate (%)	88.14	88.58	88.14	88.37
Mean Depth of Target Region (X)	102.74	103.72	124.75	138.26
Coverage of Bases≥ 1x on Target Region (%)	94	93.8	93.9	94
Coverage of Bases≥ 20x on Target Region (%)	92.5	92.6	92.8	93

Variations were using UCSC hg19 refGene (http://genome.ucsc.edu/);
The proband was the numbers II-2. The parents of proband was the number I-1 (father) and I-2 (mother). The healthy older brother of proband was the number II-1.

Supplementary Table 2. SNP Detection and Annotation in this study.

Statistics	Sample			
	I-1	I-2	II-1	II-2
Total number of variants	49674	49901	50420	51535
Number of SNPs on target	45396	45494	45887	46845
Number of known dbSNP sites on target	45145	45241	45629	46586
Synonymous SNV	9845	9828	9837	9975
Non synonymous SNV	8663	8590	8592	8785
Stopgain	68	63	60	71
Stoploss	8	7	10	7
Exonic	19994	19909	19947	20305
Intronic	21367	21553	21840	22343
Splice site	64	60	65	64
5'UTR	983	972	1006	1020
3'UTR	1256	1269	1282	1324
ncRNA exonic	969	939	962	1001
ncRNA intronic	944	966	972	1022
ncRNA splicing	2	2	2	1
Upstream	340	342	350	369
Downstream	160	165	173	163
Intergenic	1268	1254	1257	1291

Variations were using UCSC hg19 refGene (http://genome.ucsc.edu/).
The proband was the numbers II-2. The parents of proband was the number I-1 (father) and I-2 (mother). The healthy older brother of proband was the number II-1.

Supplementary Table 3. InDel Detection and Annotation in this study.

Statistics	Sample			
	I-1	I-2	II-1	II-2
Total number of InDels	4278	4407	4533	4690
Ins-coding	140	142	152	150
Del-coding	158	155	158	157
5'UTR	113	106	122	117
3'UTR	187	191	197	214
Intergenic	108	115	119	126
Total insertion	140	142	152	150
Total deletion	158	155	158	157
Heterozygous InDels	2656	2746	2832	2968
Homozygous InDels	1622	1661	1701	1722

Variations were using UCSC hg19 refGene (http://genome.ucsc.edu/);
The proband was the numbers II-2. The parents of proband was the number I-1 (father) and I-2 (mother). The healthy older brother of proband was the number II-1.

Supplementary Table 4. Characteristics of potential pathogenic mutations identified in this study.

Gene	Sample	Location	SNP ID	Nucleotide change	Predicted effect	depth/ Allele State	Type / region / protein effects	1000G/1000G.E/ESP/Exac/Exac.E/AD/AD_EAS	SIFT/PP2/MT
ARSA	I-1	chr22:51064636	rs199476360	c.925G>A	p.E309K	207/ Heterozygosity	SNP/ exonic / nonsynonymous	NA/NA/NA/ NA/NA/0.000004139 /0.00005866	D/D /D
	I-2	chr22:51064636	rs199476360	c.925G>A	p.E309K	209/ Heterozygosity	SNP/ exonic / nonsynonymous	NA/NA/NA/ NA/NA/0.000004139 /0.00005866	D/D /D
	II-1	-	-	-	-	-	-	-	-
	II-2	chr22:51064636	rs199476360	c.925G>A	p.E309K	285/ Homozygosity	SNP/ exonic / nonsynonymous	NA/NA/NA/ NA/NA/0.000004139 /0.00005866	D/D /D
SCN1A*	I-1	chr2:166911190	rs777631884	c.560G>A	p.R187Q	31/ Heterozygosity	SNP/ exonic / nonsynonymous	NA/NA/NA 0.000008332// 0.000004075 /	D/D /D
	I-2	-	-	-	-	-	-	-	-
	II-1	-	-	-	-	-	-	-	-
	II-2	chr2:166911190	rs777631884	c.560G>A	p.R187Q	56 /Heterozygosity	SNP/ exonic / nonsynonymous	NA/NA/NA 0.000008332// 0.000004075 /	D/D /D
SCN11*	I-1	-	-	-	-	-	-	-	-
	I-2	chr3:38888526	rs143537709	c.5035C>T	p.R1679	84/ Heterozygosity	SNP/ exonic / nonsynonymous	NA/NA/NA 0.00004123//0.00003257	D/D /D

								/0.00005802	
	II-1	-	-	-	-	-	-	-	-
	II-2	chr3:38 888526	rs143537 709	c.5035C> T	p.R1679	114/ Heterozygosity	SNP/ exonic / nonsynonym ous	NA/NA/NA 0.00004123//0. 00003257 /0.00005802	D/D /D
WNK1*	I-1	-	-	-	-	-	-	-	-
	I-2	chr12:1 017956	rs556506 17	c.7927T>A	p.X2643 K	70/ Heterozygosity	SNP| exonic stoploss	0.00179712/0. 0089/NA 0.00008361/0. 0012/0.000097 61 /0.0014	NA/NA /NA
	II-1	chr12:1 017956	rs556506 17	c.7927T>A	p.X2643 K	64/ Heterozygosity	SNP| exonic stoploss	0.00179712/0. 0089/NA 0.00008361/0. 0012/0.000097 61 /0.0014	NA/NA /NA
	II-2	chr12:1 017956	rs556506 17	c.7927T>A	p.X2643 K	67/ Heterozygosity	SNP| exonic stoploss	0.00179712/0. 0089/NA 0.00008361/0. 0012/0.000097 61 /0.0014	NA/NA /NA

* Genetic variation detected did not match clinical manifestations；

Nucleotide numbers are derived from cDNA ARSA sequences, GenBank accession numbers: NM_000487.5; SCN1A GenBank accession number NM_001165963; SCN11 GenBank accession number NM_014139; WNK1GenBank accession number NM_001184985.

The symbol “–” indicates data not available.

SIFT, The SIFT score (dbNSFP version 3.0) indicates the effect of the mutation on the protein sequence with the value T or D. When the mutation affects multiple protein sequences at the same time, there is a SIFT value for each protein sequence, with the minimum value. D, deleterious (sift <=0.05); T, tolerated (sift > 0.05).

PolyPhen2, PolyPhen2 was used to predict the effect of the mutation on the protein sequence based on the HumanVar database for the diagnosis of Mendelian genetic diseases (dbNSFP version 3.0). The value is D or P or B. D, probably damaging (>=0.909); P, possibly damaging (0.447<=pp2_hvar<=0.909); B, benign (pp2_hvar<=0.446).
MutationTaster, MutationTaster predicted results (dbNSFP version 3.0) indicate the effect of the mutation on the protein sequence. Mutation Taster score, with a value of 0-1. The larger the score is the more reliable the prediction. MutationTaster predicted, with values of A, D, N or P. "A" ("Disease_causing_automatic"); D"("Disease_causing"); N" ("Polymorphism"); P"("Polymorphism_automatic"). Both A and D indicate that loci may be harmful.
NA, not applicable

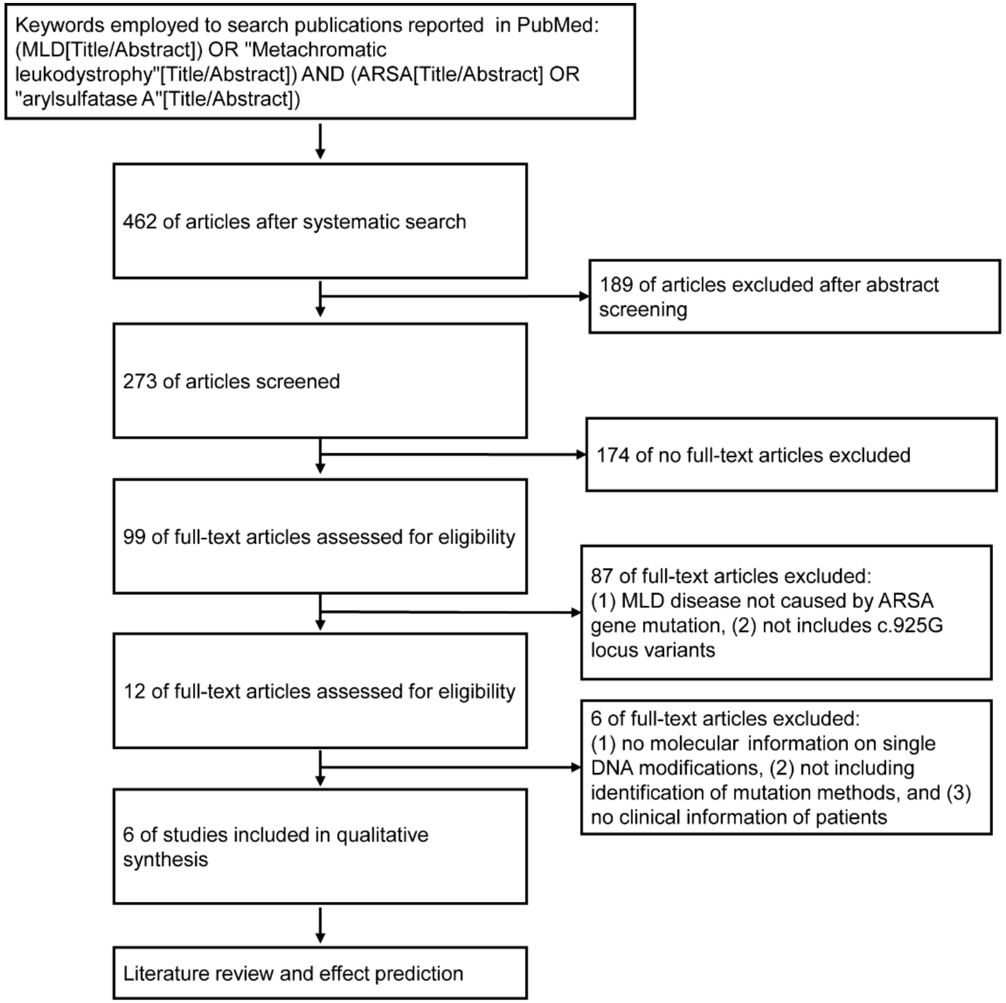

Supplementary figure 1. Flow of information through the different phases of a systematic literature review.

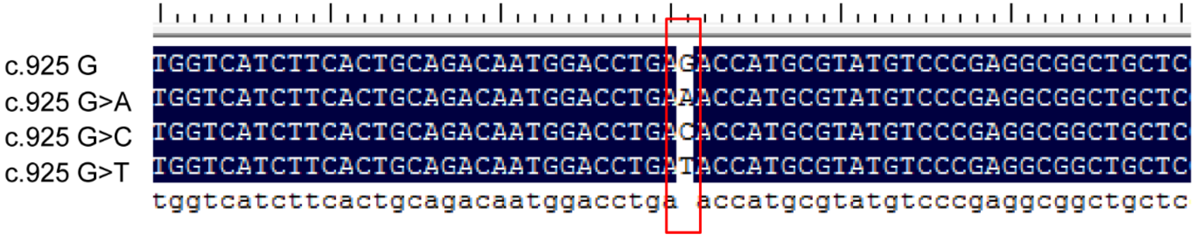

Supplementary figure 2. Wild-type ARSA cDNA /mutation vector sequences. The human wild-type ARSA cDNA cloned in the pCMV6 plasmid (c.925G). The three missense c.925G mutation (c.925G>A, c.925G>T, c.925G>C) were created on wild-type ARSA cDNA constructs, as shown in the red square. DNA sequences were confirmed using the ABI3500 sequencer. Nucleotide numbers are derived from cDNA ARSA sequences, GenBank accession numbers: NM_000487.5 and NP_000478.3.

Supplementary Table 5. Quality of the Transcriptomes in this study*

Sample	Raw reads	Raw Base (G)	Clean reads	Clean bases (G)	GC Content (%)	Q30 (%)
WT	27075342	8.12	26156665	7.845	50.705	93.825
vector	25371499	7.61	24525447	7.355	50.925	93.63
C.925 G	25047944	7.515	24284098	7.285	52.13	93.925
C.925 G>A	26867676	8.06	26161371	7.85	52.14	93.845
C.925 G>C	23929807	7.18	23161745	6.95	52.115	93.935
C.925 G>T	26868186	8.06	26217328	7.865	52.095	93.47

*Three independent experiments with average values in the table.

Supplementary Table 6. Detection of hub genes and their functional annotations

Sample	Gene	GO Slim (Ontology)	Molecular Function (GO)
c.925 G	HNF4G	[GO:0001071] nucleic acid binding transcription factor activity;[GO:0003674] molecular function;[GO:0005622] intracellular;[GO:0005575] cellular component;[GO:0003677] DNA binding;[GO:0004871] signal transducer activity;[GO:0043226] organelle;[GO:0005623] cell;[GO:0005634] nucleus;[GO:0005654] nucleoplasm;[GO:0043167] ion binding;[GO:0048856] anatomical structure development;[GO:0007165] signal transduction;[GO:0008150] biological process;[GO:0034641] cellular nitrogen compound metabolic process;[GO:0009058] biosynthetic process	GO:0003707 steroid hormone receptor activity;GO:0000978 RNA polymerase II proximal promoter sequence-specific DNA binding;GO:0000987 proximal promoter sequence-specific DNA binding
	C10orf71	NA	GO:0005515 protein binding;GO:0005488 binding;GO:0003674 molecular function
	TIMM17A	[GO:0003674] molecular function;[GO:0005575] cellular component;[GO:0005622] intracellular;[GO:0055085] transmembrane transport;[GO:0008565] protein transporter activity;[GO:0022857] transmembrane transporter activity;[GO:0006605] protein targeting;[GO:0006810] transport;[GO:0008150] biological process;[GO:0007005] mitochondrion organization;[GO:0005737] cytoplasm;[GO:0005739] mitochondrion;[GO:0043234] protein complex;[GO:0005623] cell;[GO:0005634] nucleus;[GO:0005654] nucleoplasm;[GO:0043226] organelle	GO:0015450 P-P-bond-hydrolysis-driven protein transmembrane transporter activity;GO:0008320 protein transmembrane transporter activity;GO:1904680 peptide transmembrane transporter activity
	IRF4	[GO:0005829] cytosol;[GO:0006464] cellular protein modification process;[GO:0034641] cellular nitrogen compound metabolic process;[GO:0009058] biosynthetic process;[GO:0051276] chromosome organization;[GO:0008150] biological process;[GO:0002376] immune system process;[GO:0006950] response to stress;[GO:0007155] cell adhesion;[GO:0030154] cell differentiation;[GO:0048856] anatomical	GO:0000978 RNA polymerase II proximal promoter sequence-specific DNA binding;GO:0000987 proximal promoter sequence-specific DNA binding;GO:0001228 DNA-binding

	structure development;[GO:0007165] signal transduction;[GO:0000228] nuclear chromosome;[GO:0005575] cellular component;[GO:0005694] chromosome;[GO:0043226] organelle;[GO:0005622] intracellular;[GO:0005623] cell;[GO:0005634] nucleus;[GO:0001071] nucleic acid binding transcription factor activity;[GO:0003674] molecular function;[GO:0003677] DNA binding;[GO:0043234] protein complex;[GO:0008134] transcription factor binding;[GO:0008168] methyltransferase activity;[GO:0005737] cytoplasm;[GO:0005654] nucleoplasm	transcription activator activity, RNA polymerase II-specific
SGCG	[GO:0005886] plasma membrane;[GO:0043226] organelle;[GO:0005856] cytoskeleton;[GO:0061024] membrane organization;[GO:0030154] cell differentiation;[GO:0043234] protein complex;[GO:0048856] anatomical structure development;[GO:0005575] cellular component;[GO:0005622] intracellular;[GO:0005737] cytoplasm;[GO:0005623] cell;[GO:0003674] molecular function;[GO:0008150] biological process;[GO:0003013] circulatory system process	GO:0005515 protein binding;GO:0005488 binding;GO:0003674 molecular function
EFCAB8	[GO:0043167] ion binding;[GO:0003674] molecular function	GO:0005509 calcium ion binding;GO:0046872 metal ion binding;GO:0043169 cation binding
ST6GALNAC4	[GO:0006464] cellular protein modification process;[GO:0005975] carbohydrate metabolic process;[GO:0005737] cytoplasm;[GO:0005794] Golgi apparatus;[GO:0043226] organelle;[GO:0005623] cell;[GO:0005575] cellular component;[GO:0005622] intracellular;[GO:0003674] molecular function;[GO:0016757] transferase activity, transferring glycosyl groups;[GO:0006629] lipid metabolic process;[GO:0008150] biological process;[GO:0034641] cellular nitrogen compound metabolic process;[GO:0009058] biosynthetic process	GO:0047290 (alpha-N-acetylneuraminyl-2,3-beta-galactosyl-1,3)-N-acetyl-galactosaminide 6-alpha-sialyltransferase activity;GO:0008373 sialyltransferase activity;GO:0016757 transferase activity, transferring glycosyl groups
SV2C	[GO:0003674] molecular function;[GO:0005623] cell;[GO:0005737] cytoplasm;[GO:0022857] transmembrane transporter activity;[GO:0055085] transmembrane transport;[GO:0016023] cytoplasmic membrane-bounded vesicle;[GO:0043226]	GO:0022857 transmembrane transporter activity;GO:0005215 transporter activity;GO:0005515 protein binding

	organelle;[GO:0008150] biological process;[GO:0006810] transport;[GO:0005886] plasma membrane;[GO:0005575] cellular component;[GO:0005622] intracellular	
TMEM184A	[GO:0003674] molecular function;[GO:0006810] transport;[GO:0008150] biological process;[GO:0005622] intracellular;[GO:0005575] cellular component;[GO:0005623] cell;[GO:0005768] endosome;[GO:0043226] organelle;[GO:0005737] cytoplasm	GO:0008201 heparin binding;GO:0005539 glycosaminoglycan binding;GO:1901681 sulfur compound binding
PPP1R7	[GO:0003674] molecular function;[GO:0005575] cellular component;[GO:0005622] intracellular;[GO:0005576] extracellular region;[GO:0008150] biological process;[GO:0030234] enzyme regulator activity;[GO:0006464] cellular protein modification process;[GO:0005634] nucleus;[GO:0043226] organelle;[GO:0005694] chromosome;[GO:0005737] cytoplasm;[GO:0005623] cell;[GO:0007059] chromosome segregation	GO:0019888 protein phosphatase regulator activity;GO:0019208 phosphatase regulator activity;GO:0030234 enzyme regulator activity
UTS2B	[GO:0005576] extracellular region;[GO:0005575] cellular component;[GO:0003674] molecular function;[GO:0003013] circulatory system process;[GO:0008150] biological process	GO:0005179 hormone activity;GO:0001664 G protein-coupled receptor binding;GO:0048018 receptor ligand activity
TRIM73	[GO:0003674] molecular function;[GO:0005622] intracellular;[GO:0005575] cellular component;[GO:0043167] ion binding;[GO:0005623] cell	GO:0008270 zinc ion binding;GO:0046914 transition metal ion binding;GO:0046872 metal ion binding
SEPT12	[GO:0043234] protein complex;[GO:0007049] cell cycle;[GO:0008150] biological process;[GO:0051301] cell division;[GO:0005575] cellular component;[GO:0005622] intracellular;[GO:0005623] cell;[GO:0005856] cytoskeleton;[GO:0043226] organelle;[GO:0003677] DNA binding;[GO:0003674] molecular function;[GO:0005737] cytoplasm;[GO:0043167] ion binding;[GO:0008289] lipid binding;[GO:0005929] cilium	GO:0019003 GDP binding;GO:0035091 phosphatidylinositol binding;GO:0003924 GTPase activity

	CELA2A	[GO:0008233] peptidase activity;[GO:0003674] molecular function;[GO:0005623] cell;[GO:0005737] cytoplasm;[GO:0008150] biological process;[GO:0005576] extracellular region;[GO:0005615] extracellular space;[GO:0005622] intracellular;[GO:0005575] cellular component	GO:0004252 serine-type endopeptidase activity;GO:0008236 serine-type peptidase activity;GO:0017171 serine hydrolase activity
c.925 G>A	SUN1	[GO:0003674] molecular function;[GO:0005575] cellular component;[GO:0005622] intracellular;[GO:0043226] organelle;[GO:0005635] nuclear envelope;[GO:0005634] nucleus;[GO:0005623] cell;[GO:0043234] protein complex;[GO:0007010] cytoskeleton organization;[GO:0008150] biological process;[GO:0061024] membrane organization;[GO:0005737] cytoplasm;[GO:0016023] cytoplasmic membrane-bounded vesicle;[GO:0007059] chromosome segregation;[GO:0051276] chromosome organization;[GO:0007049] cell cycle;[GO:0000003] reproduction	GO:0005521 lamin binding;GO:0043495 protein membrane anchor;GO:0005515 protein binding
	ZNF583	[GO:0003674] molecular function;[GO:0009058] biosynthetic process;[GO:0034641] cellular nitrogen compound metabolic process;[GO:0008150] biological process;[GO:0005622] intracellular;[GO:0005575] cellular component;[GO:0005623] cell;[GO:0003677] DNA binding;[GO:0001071] nucleic acid binding transcription factor activity;[GO:0005634] nucleus;[GO:0043226] organelle;[GO:0043167] ion binding	GO:0000981 DNA-binding transcription factor activity, RNA polymerase II-specific;GO:0003700 DNA-binding transcription factor activity;GO:0140110 transcription regulator activity
	TRAPPC6B	[GO:0003674] molecular function;[GO:0005575] cellular component;[GO:0005622] intracellular;[GO:0005623] cell;[GO:0005737] cytoplasm;[GO:0005794] Golgi apparatus;[GO:0043226] organelle;[GO:0005975] carbohydrate metabolic process;[GO:0008150] biological process;[GO:0005829] cytosol;[GO:0005783] endoplasmic reticulum;[GO:0006464] cellular protein modification process;[GO:0022607] cellular component assembly;[GO:0065003] macromolecular complex assembly;[GO:0006461] protein complex assembly;[GO:0006810] transport;[GO:0016192] vesicle-mediated transport;[GO:0061024] membrane organization;[GO:0009058] biosynthetic process	GO:0005515 protein binding;GO:0005488 binding;GO:0003674 molecular function

SRSF6	[GO:0003674] molecular function;[GO:0006397] mRNA processing;[GO:0008150] biological process;[GO:0022607] cellular component assembly;[GO:0022618] ribonucleoprotein complex assembly;[GO:0034641] cellular nitrogen compound metabolic process;[GO:0065003] macromolecular complex assembly;[GO:0003723] RNA binding;[GO:0009058] biosynthetic process;[GO:0006913] nucleocytoplasmic transport;[GO:0006810] transport;[GO:0005623] cell;[GO:0043226] organelle;[GO:0005654] nucleoplasm;[GO:0005634] nucleus;[GO:0008219] cell death;[GO:0006950] response to stress;[GO:0008283] cell proliferation;[GO:0048856] anatomical structure development;[GO:0030154] cell differentiation;[GO:0005622] intracellular;[GO:0005575] cellular component	GO:0036002 pre-mRNA binding;GO:0003723 RNA binding;GO:0003676 nucleic acid binding
CLTRN	[GO:0005575] cellular component;[GO:0005622] intracellular;[GO:0005576] extracellular region;[GO:0008150] biological process;[GO:0042592] homeostatic process;[GO:0006810] transport;[GO:0003674] molecular function;[GO:0005737] cytoplasm;[GO:0005623] cell;[GO:0061024] membrane organization;[GO:0016192] vesicle-mediated transport;[GO:0008233] peptidase activity;[GO:0043226] organelle;[GO:0007267] cell-cell signaling;[GO:0065003] macromolecular complex assembly;[GO:0022607] cellular component assembly;[GO:0006461] protein complex assembly;[GO:0005886] plasma membrane	GO:0042803 protein homodimerization activity;GO:0046983 protein dimerization activity;GO:0042802 identical protein binding
RIPK2	[GO:0005622] intracellular;[GO:0005575] cellular component;[GO:0004871] signal transducer activity;[GO:0043167] ion binding;[GO:0005623] cell;[GO:0005737] cytoplasm;[GO:0043234] protein complex;[GO:0006914] autophagy;[GO:0008219] cell death;[GO:0009056] catabolic process;[GO:0008283] cell proliferation;[GO:0009058] biosynthetic process;[GO:0005829] cytosol;[GO:0005856] cytoskeleton;[GO:0043226] organelle;[GO:0034641] cellular nitrogen compound metabolic process;[GO:0007165] signal transduction;[GO:0008150] biological process;[GO:0003674] molecular function;[GO:0006464] cellular protein modification process;[GO:0016301]	GO:0004706 JUN kinase kinase activity;GO:0089720 caspase binding;GO:0030274 LIM domain binding

	kinase activity;[GO:0007155] cell adhesion;[GO:0030154] cell differentiation;[GO:0048856] anatomical structure development;[GO:0002376] immune system process;[GO:0006950] response to stress	
DERL2	[GO:0008150] biological process;[GO:0040007] growth;[GO:0003674] molecular function;[GO:0005575] cellular component;[GO:0005622] intracellular;[GO:0005737] cytoplasm;[GO:0005623] cell;[GO:0005768] endosome;[GO:0043226] organelle;[GO:0008283] cell proliferation;[GO:0006950] response to stress;[GO:0006810] transport;[GO:0007165] signal transduction;[GO:0009056] catabolic process;[GO:0009058] biosynthetic process;[GO:0006457] protein folding;[GO:0006464] cellular protein modification process;[GO:0005783] endoplasmic reticulum;[GO:0005975] carbohydrate metabolic process	GO:0005515 protein binding;GO:0005488 binding;GO:0003674 molecular function
ZBTB25	[GO:0003674] molecular function;[GO:0043167] ion binding;[GO:0003677] DNA binding;[GO:0005575] cellular component;[GO:0005622] intracellular;[GO:0005623] cell;[GO:0005634] nucleus;[GO:0005737] cytoplasm;[GO:0043226] organelle;[GO:0005654] nucleoplasm;[GO:0001071] nucleic acid binding transcription factor activity;[GO:0009058] biosynthetic process;[GO:0008150] biological process;[GO:0034641] cellular nitrogen compound metabolic process	GO:0000981 DNA-binding transcription factor activity, RNA polymerase II-specific;GO:0003700 DNA-binding transcription factor activity;GO:0140110 transcription regulator activity
ZNF630	[GO:0003674] molecular function;[GO:0008150] biological process;[GO:0034641] cellular nitrogen compound metabolic process;[GO:0009058] biosynthetic process;[GO:0005575] cellular component;[GO:0005623] cell;[GO:0005622] intracellular;[GO:0003677] DNA binding;[GO:0043226] organelle;[GO:0005634] nucleus;[GO:0043167] ion binding;[GO:0001071] nucleic acid binding transcription factor activity	GO:0003677 DNA binding;GO:0046872 metal ion binding;GO:0043169 cation binding
SLC30A8	[GO:0005737] cytoplasm;[GO:0005623] cell;[GO:0005886] plasma membrane;[GO:0003674] molecular function;[GO:0043167] ion binding;[GO:0016023] cytoplasmic membrane-bounded vesicle;[GO:0043226] organelle;[GO:0007267] cell-cell signaling;[GO:0042592] homeostatic process;[GO:0016192] vesicle-	GO:0005385 zinc ion transmembrane transporter activity;GO:0072509 divalent inorganic cation transmembrane transporter activity;GO:0046915 transition metal

	mediated transport;[GO:0055085] transmembrane transport;[GO:0005575] cellular component;[GO:0022857] transmembrane transporter activity;[GO:0005622] intracellular;[GO:0006810] transport;[GO:0008150] biological process;[GO:0006950] response to stress;[GO:0002376] immune system process	ion transmembrane transporter activity
CENPS-CORT	[GO:0003674] molecular function;[GO:0005576] extracellular region;[GO:0005575] cellular component	
ATP5MC2	[GO:0005622] intracellular;[GO:0005575] cellular component;[GO:0005623] cell;[GO:0043226] organelle;[GO:0043234] protein complex;[GO:0005737] cytoplasm;[GO:0005739] mitochondrion;[GO:0008289] lipid binding;[GO:0022857] transmembrane transporter activity;[GO:0055085] transmembrane transport;[GO:0006810] transport;[GO:0006091] generation of precursor metabolites and energy;[GO:0008150] biological process;[GO:0034641] cellular nitrogen compound metabolic process;[GO:0044281] small molecule metabolic process;[GO:0009058] biosynthetic process;[GO:0003674] molecular function	GO:0046933 proton-transporting ATP synthase activity, rotational mechanism;GO:0044769 ATPase activity, coupled to transmembrane movement of ions, rotational mechanism;GO:0019829 cation-transporting ATPase activity
PHOSPHO2	[GO:0003674] molecular function;[GO:0043167] ion binding;[GO:0008150] biological process;[GO:0016791] phosphatase activity	GO:0033883 pyridoxal phosphatase activity;GO:0016791 phosphatase activity;GO:0042578 phosphoric ester hydrolase activity
IL12B	[GO:0005623] cell;[GO:0005737] cytoplasm;[GO:0009058] biosynthetic process;[GO:0050877] neurological system process;[GO:0043234] protein complex;[GO:0044403] symbiosis, encompassing mutualism through parasitism;[GO:0040011] locomotion;[GO:0048870] cell motility;[GO:0040007] growth;[GO:0007165] signal transduction;[GO:0006810] transport;[GO:0006913] nucleocytoplasmic transport;[GO:0006605] protein targeting;[GO:0008219] cell death;[GO:0007049] cell cycle;[GO:0003674] molecular function;[GO:0002376] immune system process;[GO:0008283] cell proliferation;[GO:0006464] cellular protein modification	GO:0042164 interleukin-12 alpha subunit binding;GO:0045519 interleukin-23 receptor binding;GO:0019972 interleukin-12 binding

	COG5	process;[GO:0006950] response to stress;[GO:0007155] cell adhesion;[GO:0030154] cell differentiation;[GO:0048856] anatomical structure development;[GO:0000003] reproduction;[GO:0008150] biological process;[GO:0005575] cellular component;[GO:0005622] intracellular;[GO:0004871] signal transducer activity;[GO:0005576] extracellular region;[GO:0005615] extracellular space [GO:0005634] nucleus;[GO:0005654] nucleoplasm;[GO:0016192] vesicle-mediated transport;[GO:0008150] biological process;[GO:0006810] transport;[GO:0043234] protein complex;[GO:0003674] molecular function;[GO:0005829] cytosol;[GO:0005622] intracellular;[GO:0043226] organelle;[GO:0005623] cell;[GO:0005737] cytoplasm;[GO:0005794] Golgi apparatus;[GO:0005575] cellular component	GO:0005515 protein binding;GO:0005488 binding;GO:0003674 molecular function
	MTCP1	[GO:0005622] intracellular;[GO:0005575] cellular component;[GO:0005623] cell;[GO:0005737] cytoplasm;[GO:0043226] organelle;[GO:0005739] mitochondrion;[GO:0008283] cell proliferation;[GO:0008150] biological process	GO:0043539 protein serine/threonine kinase activator activity;GO:0030295 protein kinase activator activity;GO:0019209 kinase activator activity
c.925 G>C	CFAP126	[GO:0003674] molecular function;[GO:0005623] cell;[GO:0005737] cytoplasm;[GO:0005886] plasma membrane;[GO:0005929] cilium;[GO:0005815] microtubule organizing center;[GO:0005856] cytoskeleton;[GO:0043226] organelle;[GO:0008150] biological process;[GO:0005622] intracellular;[GO:0005575] cellular component	
	F5	[GO:0043167] ion binding;[GO:0005615] extracellular space;[GO:0005576] extracellular region;[GO:0005575] cellular component;[GO:0005622] intracellular;[GO:0005623] cell;[GO:0005737] cytoplasm;[GO:0005794] Golgi apparatus;[GO:0043226] organelle;[GO:0008150] biological process;[GO:0006810] transport;[GO:0016192] vesicle-mediated transport;[GO:0003013] circulatory system process;[GO:0003674] molecular function;[GO:0009058] biosynthetic process;[GO:0061024] membrane organization;[GO:0006950] response to stress;[GO:0016023] cytoplasmic membrane-bounded vesicle;[GO:0005783] endoplasmic reticulum;[GO:0006464] cellular protein modification process;[GO:0022607] cellular component assembly;[GO:0006461] protein	GO:0005507 copper ion binding;GO:0046914 transition metal ion binding;GO:0046872 metal ion binding

	complex assembly;[GO:0065003] macromolecular complex assembly;[GO:0005886] plasma membrane;[GO:0005975] carbohydrate metabolic process	
LRMP	[GO:0002376] immune system process;[GO:0005783] endoplasmic reticulum;[GO:0005815] microtubule organizing center;[GO:0005886] plasma membrane;[GO:0005575] cellular component;[GO:0005622] intracellular;[GO:0005623] cell;[GO:0005856] cytoskeleton;[GO:0043226] organelle;[GO:0008150] biological process;[GO:0000003] reproduction;[GO:0016192] vesicle-mediated transport;[GO:0061024] membrane organization;[GO:0006810] transport;[GO:0005634] nucleus;[GO:0005694] chromosome;[GO:0005635] nuclear envelope;[GO:0005737] cytoplasm	
MTRNR2L4	[GO:0005737] cytoplasm;[GO:0005623] cell;[GO:0005575] cellular component;[GO:0005622] intracellular;[GO:0005576] extracellular region	GO:0048019 receptor antagonist activity;GO:0030547 receptor inhibitor activity;GO:0030545 receptor regulator activity
ELP4	[GO:0005737] cytoplasm;[GO:0005622] intracellular;[GO:0005623] cell;[GO:0005634] nucleus;[GO:0005654] nucleoplasm;[GO:0043226] organelle;[GO:0043234] protein complex;[GO:0005575] cellular component;[GO:0003674] molecular function;[GO:0019899] enzyme binding;[GO:0016746] transferase activity, transferring acyl groups;[GO:0006464] cellular protein modification process;[GO:0008150] biological process;[GO:0030234] enzyme regulator activity;[GO:0051276] chromosome organization;[GO:0034641] cellular nitrogen compound metabolic process;[GO:0009058] biosynthetic process	GO:0008607 phosphorylase kinase regulator activity;GO:0000993 RNA polymerase II complex binding;GO:0043175 RNA polymerase core enzyme binding
IL5RA	[GO:0030154] cell differentiation;[GO:0048856] anatomical structure development;[GO:0000902] cell morphogenesis;[GO:0008150] biological process;[GO:0006464] cellular protein modification process;[GO:0007165] signal transduction;[GO:0005622] intracellular;[GO:0005615] extracellular space;[GO:0005576] extracellular region;[GO:0005575] cellular component;[GO:0004871] signal transducer activity;[GO:0005886] plasma membrane;[GO:0005623] cell;[GO:0002376] immune system process;[GO:0006950] response to stress;[GO:0003674] molecular function;[GO:0040011] locomotion;[GO:0008283] cell proliferation	GO:0004914 interleukin-5 receptor activity;GO:0004896 cytokine receptor activity;GO:0019955 cytokine binding

CALCR	[GO:0003674] molecular function;[GO:0004871] signal transducer activity;[GO:0008150] biological process;[GO:0007165] signal transduction;[GO:0005575] cellular component;[GO:0005622] intracellular;[GO:0005623] cell;[GO:0005737] cytoplasm;[GO:0008565] protein transporter activity;[GO:0016192] vesicle-mediated transport;[GO:0042592] homeostatic process;[GO:0006810] transport;[GO:0007009] plasma membrane organization;[GO:0061024] membrane organization;[GO:0044281] small molecule metabolic process;[GO:0034641] cellular nitrogen compound metabolic process;[GO:0009058] biosynthetic process;[GO:0005829] cytosol;[GO:0005886] plasma membrane;[GO:0016023] cytoplasmic membrane-bounded vesicle;[GO:0043226] organelle;[GO:0002376] immune system process;[GO:0048856] anatomical structure development;[GO:0030154] cell differentiation;[GO:0005929] cilium	GO:0032841 calcitonin binding;GO:0001635 calcitonin gene-related peptide receptor activity;GO:0097643 amylin receptor activity
AAK1	[GO:0008150] biological process;[GO:0043167] ion binding;[GO:0016301] kinase activity;[GO:0006464] cellular protein modification process;[GO:0003674] molecular function;[GO:0016192] vesicle-mediated transport;[GO:0007165] signal transduction;[GO:0006810] transport;[GO:0043226] organelle;[GO:0016023] cytoplasmic membrane-bounded vesicle;[GO:0005622] intracellular;[GO:0005575] cellular component;[GO:0005886] plasma membrane;[GO:0005737] cytoplasm;[GO:0005623] cell	GO:0035612 AP-2 adaptor complex binding;GO:0005112 Notch binding;GO:0004674 protein serine/threonine kinase activity
SRSF9	[GO:0005575] cellular component;[GO:0005622] intracellular;[GO:0005634] nucleus;[GO:0005623] cell;[GO:0043226] organelle;[GO:0005730] nucleolus;[GO:0005654] nucleoplasm;[GO:0003674] molecular function;[GO:0006397] mRNA processing;[GO:0008150] biological process;[GO:0022607] cellular component assembly;[GO:0022618] ribonucleoprotein complex assembly;[GO:0034641] cellular nitrogen compound metabolic process;[GO:0065003] macromolecular complex assembly;[GO:0003723] RNA binding;[GO:0006810] transport;[GO:0009058] biosynthetic process;[GO:0006913] nucleocytoplasmic transport	GO:0019904 protein domain specific binding;GO:0003723 RNA binding;GO:0003676 nucleic acid binding

SMIM24	[GO:0043226] organelle;[GO:0008150] biological process;[GO:0003674] molecular function;[GO:0005575] cellular component;[GO:0005576] extracellular region	GO:0003674 molecular function
ZC3H12D	[GO:0004518] nuclease activity;[GO:0003674] molecular function;[GO:0040007] growth;[GO:0007049] cell cycle;[GO:0008150] biological process;[GO:0005575] cellular component;[GO:0005622] intracellular;[GO:0005623] cell;[GO:0005737] cytoplasm;[GO:0043226] organelle;[GO:0005634] nucleus;[GO:0043167] ion binding;[GO:0034641] cellular nitrogen compound metabolic process	GO:0004519 endonuclease activity;GO:0004518 nuclease activity;GO:0016788 hydrolase activity, acting on ester bonds
STT3A	[GO:0005575] cellular component;[GO:0009058] biosynthetic process;[GO:0006464] cellular protein modification process;[GO:0005975] carbohydrate metabolic process;[GO:0008150] biological process;[GO:0003674] molecular function;[GO:0016757] transferase activity, transferring glycosyl groups;[GO:0005622] intracellular;[GO:0005623] cell;[GO:0005737] cytoplasm;[GO:0005783] endoplasmic reticulum;[GO:0043226] organelle;[GO:0043234] protein complex	GO:0004579 dolichyl-diphosphooligosaccharide-protein glycotransferase activity;GO:0004576 oligosaccharyl transferase activity;GO:0016758 transferase activity, transferring hexosyl groups
ACTL6A	[GO:0005886] plasma membrane;[GO:0006464] cellular protein modification process;[GO:0051276] chromosome organization;[GO:0006259] DNA metabolic process;[GO:0006950] response to stress;[GO:0034641] cellular nitrogen compound metabolic process;[GO:0009058] biosynthetic process;[GO:0007165] signal transduction;[GO:0040007] growth;[GO:0006810] transport;[GO:0006605] protein targeting;[GO:0048856] anatomical structure development;[GO:0005575] cellular component;[GO:0005622] intracellular;[GO:0005623] cell;[GO:0005634] nucleus;[GO:0005654] nucleoplasm;[GO:0043226] organelle;[GO:0043234] protein complex;[GO:0008150] biological process;[GO:0009056] catabolic process;[GO:0006914] autophagy;[GO:0007005] mitochondrion organization;[GO:0005694] chromosome;[GO:0000228] nuclear chromosome;[GO:0000988] transcription factor activity, protein binding;[GO:0003674] molecular function;[GO:0003677] DNA binding	GO:0000980 RNA polymerase II distal enhancer sequence-specific DNA binding;GO:0031492 nucleosomal DNA binding;GO:0001158 enhancer sequence-specific DNA binding

	IFIH1	[GO:0005575] cellular component;[GO:0005622] intracellular;[GO:0043167] ion binding;[GO:0003674] molecular function;[GO:0008219] cell death;[GO:0044403] symbiosis, encompassing mutualism through parasitism;[GO:0005829] cytosol;[GO:0006464] cellular protein modification process;[GO:0005737] cytoplasm;[GO:0005623] cell;[GO:0005634] nucleus;[GO:0043226] organelle;[GO:0008150] biological process;[GO:0002376] immune system process;[GO:0006950] response to stress;[GO:0007165] signal transduction;[GO:0003723] RNA binding;[GO:0003677] DNA binding;[GO:0004386] helicase activity	GO:0003725 double-stranded RNA binding;GO:0003727 single-stranded RNA binding;GO:0004386 helicase activity
c.925 G>T	BCL2L1	[GO:0006810] transport;[GO:0061024] membrane organization;[GO:0007009] plasma membrane organization;[GO:0009056] catabolic process;[GO:0006950] response to stress;[GO:0016192] vesicle-mediated transport;[GO:0006914] autophagy;[GO:0016023] cytoplasmic membrane-bounded vesicle;[GO:0044403] symbiosis, encompassing mutualism through parasitism;[GO:0019899] enzyme binding;[GO:0043234] protein complex;[GO:0005575] cellular component;[GO:0005622] intracellular;[GO:0007049] cell cycle;[GO:0008150] biological process;[GO:0051301] cell division;[GO:0005623] cell;[GO:0005737] cytoplasm;[GO:0043226] organelle;[GO:0005635] nuclear envelope;[GO:0005739] mitochondrion;[GO:0005634] nucleus;[GO:0002376] immune system process;[GO:0003674] molecular function;[GO:0007005] mitochondrion organization;[GO:0007165] signal transduction;[GO:0008219] cell death;[GO:0005829] cytosol;[GO:0005815] microtubule organizing center;[GO:0005856] cytoskeleton;[GO:0008283] cell proliferation;[GO:0030154] cell differentiation;[GO:0040007] growth;[GO:0000003] reproduction;[GO:0009790] embryo development;[GO:0048856] anatomical structure development	GO:0051434 BH3 domain binding;GO:0051400 BH domain binding;GO:0070513 death domain binding
	S100A4	[GO:0005575] cellular component;[GO:0043167] ion binding;[GO:0005576] extracellular region;[GO:0005615] extracellular space;[GO:0005622] intracellular;[GO:0008150] biological process;[GO:0048856] anatomical structure development;[GO:0030154]	GO:0050786 RAGE receptor binding;GO:0048306 calcium-dependent protein binding;GO:0003779 actin binding

PSMC5	cell differentiation;[GO:0000902] cell morphogenesis;[GO:0003723] RNA binding;[GO:0003674] molecular function;[GO:0043226] organelle;[GO:0005634] nucleus;[GO:0005623] cell;[GO:0005737] cytoplasm;[GO:0007165] signal transduction;[GO:0008092] cytoskeletal protein binding [GO:0003674] molecular function;[GO:0043167] ion binding;[GO:0008134] transcription factor binding;[GO:0009056] catabolic process;[GO:0006520] cellular amino acid metabolic process;[GO:0016887] ATPase activity;[GO:0044403] symbiosis, encompassing mutualism through parasitism;[GO:0008219] cell death;[GO:0040011] locomotion;[GO:0002376] immune system process;[GO:0006464] cellular protein modification process;[GO:0007165] signal transduction;[GO:0005575] cellular component;[GO:0005622] intracellular;[GO:0005623] cell;[GO:0043234] protein complex;[GO:0000902] cell morphogenesis;[GO:0048856] anatomical structure development;[GO:0030154] cell differentiation;[GO:0000988] transcription factor activity, protein binding;[GO:0007049] cell cycle;[GO:0008150] biological process;[GO:0006950] response to stress;[GO:0044281] small molecule metabolic process;[GO:0034641] cellular nitrogen compound metabolic process;[GO:0022607] cellular component assembly;[GO:0006461] protein complex assembly;[GO:0065003] macromolecular complex assembly;[GO:0009058] biosynthetic process;[GO:0005829] cytosol;[GO:0005576] extracellular region;[GO:0005615] extracellular space;[GO:0005737] cytoplasm;[GO:0005634] nucleus;[GO:0043226] organelle;[GO:0005654] nucleoplasm	GO:0031531 thyrotropin-releasing hormone receptor binding;GO:0051428 peptide hormone receptor binding;GO:0017025 TBP-class protein binding
GNG11	[GO:0006091] generation of precursor metabolites and energy;[GO:0008150] biological process;[GO:0005886] plasma membrane;[GO:0043234] protein complex;[GO:0005623] cell;[GO:0004871] signal transducer activity;[GO:0005575] cellular component;[GO:0005622] intracellular;[GO:0007165] signal	GO:0031681 G-protein beta-subunit binding;GO:0003924 GTPase activity;GO:0017111 nucleoside-triphosphatase activity

	transduction;[GO:0044281] small molecule metabolic process;[GO:0003674] molecular function;[GO:0003924] GTPase activity	
OTUD5	[GO:0008233] peptidase activity;[GO:0008150] biological process;[GO:0006464] cellular protein modification process;[GO:0003674] molecular function;[GO:0006950] response to stress;[GO:0005829] cytosol;[GO:0005737] cytoplasm;[GO:0005623] cell;[GO:0005622] intracellular;[GO:0005575] cellular component;[GO:0002376] immune system process	GO:0061578 Lys63-specific deubiquitinase activity;GO:1990380 Lys48-specific deubiquitinase activity;GO:0004843 thiol-dependent ubiquitin-specific protease activity
CLEC16A	[GO:0005764] lysosome;[GO:0005768] endosome;[GO:0006914] autophagy;[GO:0008150] biological process;[GO:0043226] organelle;[GO:0005575] cellular component;[GO:0005622] intracellular;[GO:0005623] cell;[GO:0005737] cytoplasm;[GO:0005773] vacuole;[GO:0003674] molecular function	GO:0017137 Rab GTPase binding;GO:0017016 Ras GTPase binding;GO:0031267 small GTPase binding
CCDC116	[GO:0003674] molecular function;[GO:0005622] intracellular;[GO:0005575] cellular component;[GO:0005815] microtubule organizing center;[GO:0005856] cytoskeleton;[GO:0043226] organelle;[GO:0005737] cytoplasm;[GO:0005623] cell	GO:0005515 protein binding;GO:0005488 binding;GO:0003674 molecular function
NOP53	[GO:0008150] biological process;[GO:0003674] molecular function;[GO:0003723] RNA binding;[GO:0005634] nucleus;[GO:0043226] organelle;[GO:0005730] nucleolus;[GO:0005623] cell;[GO:0005575] cellular component;[GO:0005622] intracellular	GO:0008097 5S rRNA binding;GO:0002039 p53 binding;GO:0019843 rRNA binding

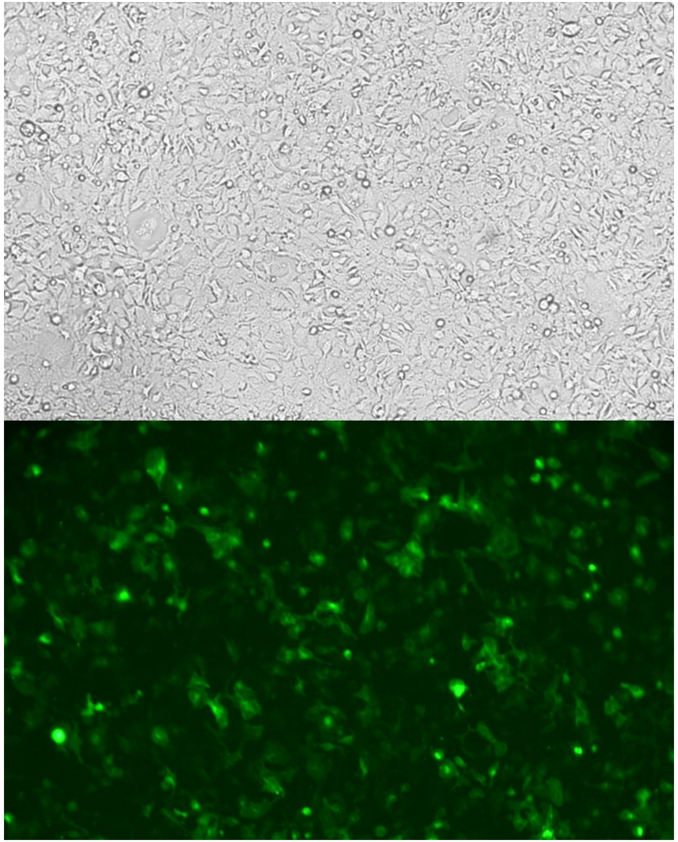

Supplementary figure 3. Fluorescence of HEK293 cells transfected with pCMV6-GFP fusion plasmid verified the efficiency of transfection. HEK293 cells were seeded 24 hours before transfection and were collected 48 hours after the transfection. The ratio of the efficiency of transfection was detected by fluorescence of HEK293 cells transfected with pCMV6-GFP fusion plasmid.

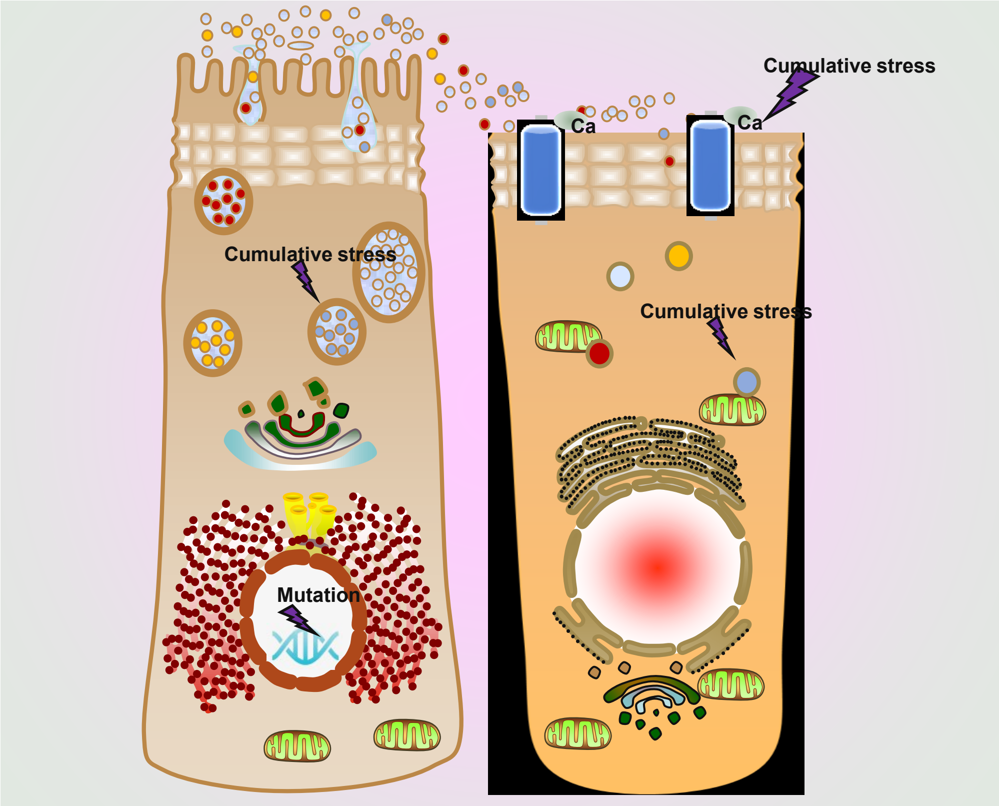

Supplementary figure 4. The defect in arylsulfatase A deficiency. ARSA gene c.925G three mutation (c. 925G>A, c. 925G>T, c. 925G>C) result in the production of defective the arylsulfatase A enzyme. It is speculated that the mutation in this region will cause the change of disulfide bond, lead to accumulation pressure, and affect the calcium ion binding and the structure of mitochondria and other related organelle membranes.
